# Using the Correlation Intensity Index to Build a Model of Cardiotoxicity of Piperidine Derivatives

**DOI:** 10.3390/molecules28186587

**Published:** 2023-09-12

**Authors:** Alla P. Toropova, Andrey A. Toropov, Alessandra Roncaglioni, Emilio Benfenati

**Affiliations:** Istituto di Ricerche Farmacologiche Mario Negri IRCCS, Department of Environmental, Health Science, Via Mario Negri 2, 20156 Milano, Italy; andrey.toropov@marionegri.it (A.A.T.); alessandra.roncaglioni@marionegri.it (A.R.); emilio.benfenati@marionegri.it (E.B.)

**Keywords:** cardiotoxicity, Monte Carlo method, computational chemistry, correlation intensity index, CORAL software

## Abstract

The assessment of cardiotoxicity is a persistent problem in medicinal chemistry. Quantitative structure–activity relationships (QSAR) are one possible way to build up models for cardiotoxicity. Here, we describe the results obtained with the Monte Carlo technique to develop hybrid optimal descriptors correlated with cardiotoxicity. The predictive potential of the cardiotoxicity models (*pIC*50, Ki in nM) of piperidine derivatives obtained using this approach provided quite good determination coefficients for the external validation set, in the range of 0.90–0.94. The results were best when applying the so-called correlation intensity index, which improves the predictive potential of a model.

## 1. Introduction

The risk of developing cardiotoxicity against the background of treating carcinogenic pathologies is one of the most urgent problems of modern oncology and cardiology. Piperidine derivatives are of exceptional interest due to their potential biological activity, such as antiviral, antibacterial, antitumor, and many others [1].

Current anticancer therapy includes many drugs with various mechanisms and a spectrum of actions. One of the most important groups in this number of drugs are antibiotics with antitumor activity, which play a massive role in the chemotherapy of various oncological diseases [2]. Their effectiveness has been clinically proven. However, despite the favorable course of the disease, when using this group of drugs, patients experience a number of undesirable side effects from various organs and systems that can develop not only during therapy but also after its completion. One of the main side effects is cardiotoxicity. This term includes various adverse events from the cardiovascular system against the background of drug therapy for oncological diseases. Such manifestations of cardiotoxicity as pain in the heart, blood pressure, heart rhythm disturbances, myocarditis, pericarditis, and heart attacks reduce a patient’s quality of life. Still, sometimes they become serious reasons for discontinuing or not prescribing the drug. For some medicines in this group, for example, alkylating agents, cardiotoxicity is a limiting factor.

Antibiotics with antitumor activity currently occupy a leading place in the treatment of oncological diseases [3]; as a result, the correction of their side effects, particularly cardiotoxicity, remains one of the most urgent problems for oncologists, cardiologists, and general practitioners. The frequency of development of various dysfunctions of the heart reaches greater values. At the same time, both reversible and irreversible consequences are quite dangerous. Prevention and treatment of cardiotoxicity remain mandatory, but complicated clinical tasks for a doctor due to the irreversibility and progressive nature of the disease changes most in the functioning of the cardiovascular system. Cardiotoxicity complications significantly impair patients’ quality of life and reduce the duration of life, and mortality from cardiovascular diseases still globally ranks first [4].

In addition, psychopharmacology and psychopharmacotherapy of depressive states are dynamically developing areas, and antidepressants are the second most prescribed drugs among all psychotropic drugs [5]. Such a high rating of these psychotropic drugs is because about 5% of the world’s population suffers from depression (according to WHO). However, high doses and long-term use of medications in this group lead to cardiotoxic effects. The cardiotoxicity of tricyclic antidepressants is manifested with conduction disturbances in the atrioventricular node and ventricles of the heart (quinine-like action), arrhythmias, and a decrease in myocardial contractility. Doxepin and amoxapine have the least cardiotoxicity. Treatment of patients with cardiovascular pathology with tricyclic antidepressants should be carefully monitored, and high doses should not be used.

Cardiovascular diseases such as coronary artery disease, valvular heart disease, arrhythmias, and heart failure are serious health risks and often require lifelong treatment. Much attention is paid to the psychological consequences of life for people with cardiac diseases [6]. Anxiety symptoms are common among patients with cardiovascular diseases and may worsen the prognosis for these patients. Symptoms of anxiety and depression can prevent lifestyle changes and adherence to therapy, as well as reduce the effectiveness of cardiac rehabilitation.

There is increasing evidence of the widespread use of psychotropic drugs in cardiac patients for comorbid psychiatric disorders [7]. The side effects of psychotropic medications on the part of the cardiovascular system include disturbances in the rhythm and conduction of the heart. For example, recent studies have shown that antidepressants were associated with increased mortality and an altered beta-blocker effect in patients with heart failure. In addition, the use of antipsychotic drugs in patients after acute myocardial infarction is necessary [6,7].

An increase in morbidity and mortality under the influence of depression in patients with cardiovascular diseases dictates the imperative need for preliminary analysis of both drugs’ therapeutic efficacy and cardiotoxic potential [8]. In other words, when developing a treatment plan for a depressed patient with heart disease, one should carefully weigh any intervention’s risk/benefit ratio. However, the choice of antidepressants is complicated because many can cause cardiovascular side effects, such as orthostatic hypotension, hypertension, and impaired cardiac conduction. In addition, clinically significant drug interactions should be considered when choosing treatment. Unfortunately, the number of clinical studies explicitly investigating the safety of antidepressants in patients with cardiovascular disease is limited, and the studies that are conducted have generally included a small number of patients.

The human ether-a-go-go-related gene (hERG) potassium channel plays a pivotal role in cardiac rhythm regulation, and the cardiotoxicity data associated with hERG inhibition using drugs and environmental chemicals provides important information for medicinal chemistry [9,10]. As stated above, cardiac problems are among the most complex in medicine [11], and there is a clear trend toward them becoming more important [12]. Identifying potential human ether-a-go-go-related gene (hERG) potassium channel blockers is an important part of drug discovery and checking up on drug safety processes in pharmaceutical industries and academic drug discovery centers [10]. The most popular idea at present is considered to begin the corresponding searches with the choice of idealization (a certain success), that is a molecule that absorbs the preferred qualities in the complete form. In order to prioritize molecules during the early drug discovery phase and to reduce the risk of the necessity of an additional preliminary checking-up of pharmaceutical agents, computational approaches have been developed to predict the potential of hERG blockage of new drug candidates. In other words, estimating the cardiac toxicity of organic hERG blockers is an important theoretical and practical task of medicinal chemistry. Potential hERG inhibitors must be identified for drug discovery and safety [13]; however, the experimental analysis of all potential hERG inhibitors is impossible because there are so many of them. 

Computational models for the cardiac toxicity of organic hERG blockers are an attractive alternative to real experiments [14]. Quantitative structure–activity relationships (QSAR) are common computational approaches [15]. Such models can be obtained using machine learning based on graph theory, support vector machine, random forest, artificial neural networks, and other approaches [16,17].

CORAL software (http://www.insilico.eu/coral, accessed on 1 September 2023) is a tool for building up QSAR models for various endpoints with the Monte Carlo method [18,19,20,21,22,23,24,25,26,27]. The CORAL software was recently updated with what is called the index of ideality of correlation (*IIC*) [28] and the correlation intensity index (*CII*) [29]. *IIC* and *CII* are indicators of the predictive potential of QSAR. 

*IIC* differs from other criteria of the statistical quality of linear regression models with a unique ability since it is a measure that is sensitive both to the value of the correlation coefficient and to the value of the mean absolute error (MAE).

In principle, *CII* has some analogy with the known cross-validation measures, but this analogy is partial. While the traditional cross-validated test is based on averaging the difference between the correlation coefficient before and after the “removing” of molecules from the set (training, calibration, or testing), the *CII* considers the average value of the difference observed removing only molecules which reduce the correlation coefficient in the set.

Here, the ability to improve the predictive potential of cardiotoxicity models using the *IIC* and *CII* is studied.

## 2. Results 

### 2.1. QSAR Models Based on TF_1_

The Monte Carlo optimization with the target function *TF*_1_ for three random splits (#1, #2, and #3) provides the following models:

*pIC*50 = 3.456(±0.058) + 0.0632(±0.0019) × *DCW*(1, 15) (1)

*pIC*50 = 4.082(±0.019) + 0.1498(±0.0025) × *DCW*(1, 15) (2)

*pIC*50 = 2.618(±0.140) + 0.1654(±0.0090) × *DCW*(1, 35) (3)

Table 1 provides the statistical quality of these models.

### 2.2. QSAR Models Based on TF_2_

The Monte Carlo optimization with the target function *TF*_2_ for three random splits (#1, #2, and #3) provides the following models:

*pIC*50 = 3.202(±0.068) + 0.0867 (±0.0027) × *DCW*(1, 15) (4)

*pIC*50 = 3.036 (±0.084) + 0.0730 (±0.0028) × *DCW*(1, 15) (5)

*pIC*50 = 3.490(±0.077) + 0.0803 (±0.0035) × *DCW*(1, 15) (6)

Table 2 provides the statistical quality of these models. The average value of the coefficient of determination for these models is about 0.6 (for the set as a whole). However, there is a paradox described earlier [28]. The influence of the *IICc* leads to an improvement in the coefficient of determination for the calibration and the validation sets but not to the detriment of training sets, where the coefficient of determination is lower.

**Figure 1:**
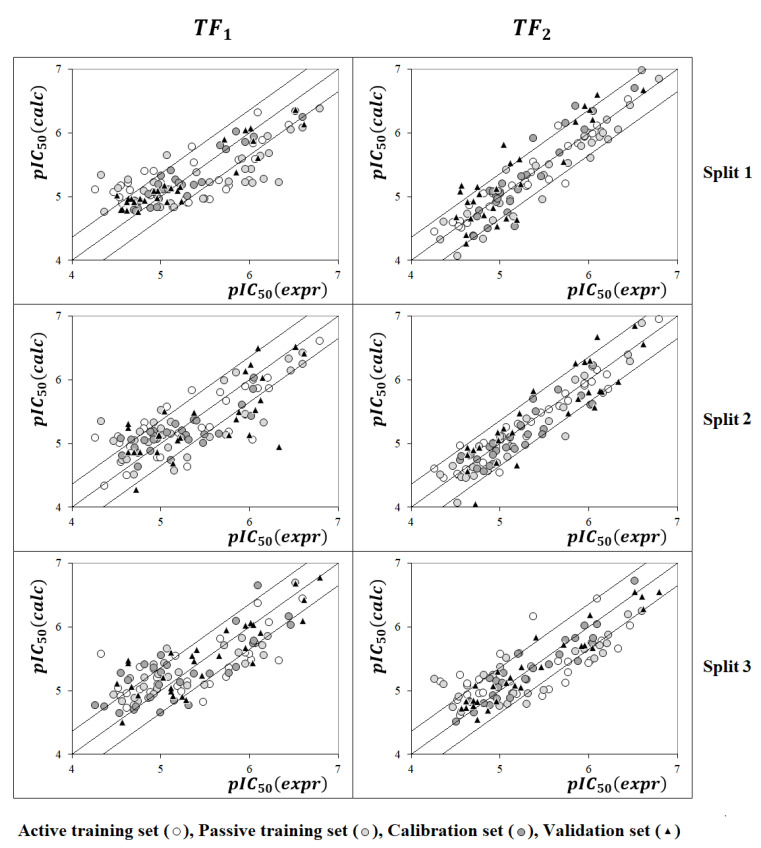
Examples of models calculated using target functions *TF*_1_ and *TF*_2_.

**Figure 2:**
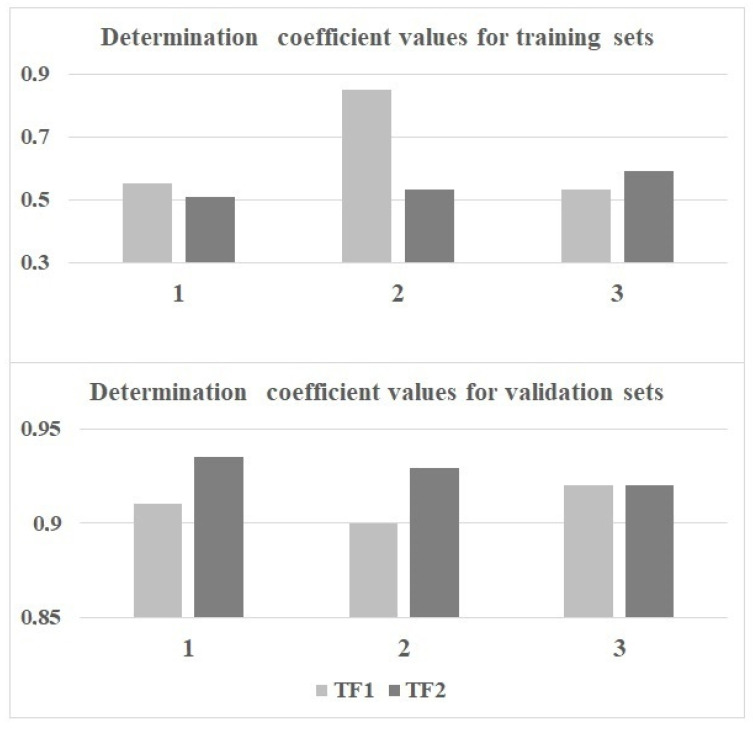
The comparison of determination coefficients for the active training and the validation sets using *TF*_1_ and *TF*_2_ for splits 1–3.

Figure 1 compares models calculated with the target functions *TF*_1_ and *TF*_2_. Models calculated using *TF*_2_ are preferred since Figure 2 confirms that the average determination coefficient values of *TF*_2_-models are larger than those of *TF*_1_-models for all three random splits. 

**Figure 3:**
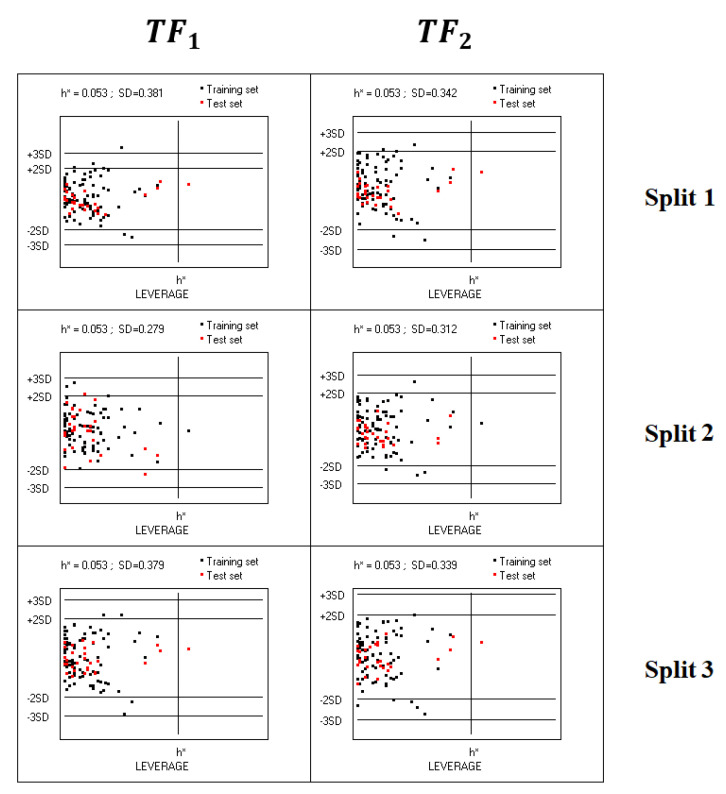
Williams plots of models calculated using target functions *TF*_1_ and *TF*_2_ for splits 1–3. The training set is the union of the active and passive training sets together with the calibration set; the compounds of the external test set are indicated in red.

Williams plots for all considered models indicated that there are practically no outliers for both *TF*_1_-models and *TF*_2_-models (Figure 3).

## 3. Discussion 

The models’ advantage is their user-friendliness since their implementation requires only SMILES and numerical data for an endpoint without any other descriptors. There are special rules to define the mechanistic interpretation as well as the applicability domain. The described approach provides models following OECD principles [30,31]. The main essence of the above document concentrated on the well-known five OECD principles [26,30,31,32] is descripted below:

- A defined endpoint;
- An unambiguous algorithm;
- A defined applicability domain;
- Appropriate measures of goodness-of-fit, robustness, and predictivity;
- A mechanistic interpretation, if possible.

Table 3 lists the molecular features of statistically stable promoters of an increase or decrease of the *pIC*50. These data are selected according to the following:

(i) Molecular features extracted from SMILES or HSG with significant prevalence in the training and calibration sets;

(ii) Molecular features which have positive correlation weights (CW) for all three runs of the Monte Carlo optimization; 

(iii) Molecular features with negative CW for all three runs of the Monte Carlo optimization.

There are stable promoters of the *pIC*50 increase related to all distributions. For instance, the promoters of an increase of *pIC*50 are the presence of nitrogen connected with carbon when Morgan extended connectivity of carbon atoms is equal to 5, 6, and 7 or Morgan extended connectivity of nitrogen atoms equals 4. In contrast, promoters of decrease of *pIC*50 are vertex degrees of carbon atoms equal to 2 or 3 and degrees of nitrogen atoms equal to 2. Some other features become promoters of an increase or decrease of cardiotoxicity (Table 3). 

**Figure 4:**
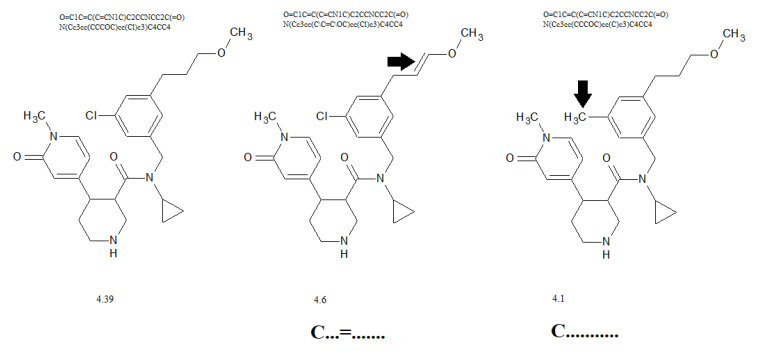
The influence of presence/absence of promoters on the calculated cardiotoxicity.

Figure 4 contains examples of influence promoters of increase (C…=…….) and decrease (C………..) to the calculated cardiotoxicity values.

The comparison of the statistical quality of the models using the target functions *TF*_1_ and *TF*_2_ presented in Table 1 and Table 2 indicates that *TF*_2_ provides better results. 

Table 4 contains the comparison of models for cardiotoxicity suggested in the literature.

The best model is observed for *TF*_1_ (split-1); however, the results for the other two splits in the case of the *TF*_1_-model are worse, and the variance of the coefficient of determination for the validation set is significant. In contrast, the average value of the coefficient of determination for the validation set in the case of the *TF*_2_-model is more significant, and the variance is less than those in the case of the *TF*_1_-model. Thus, despite the excellent result for split-1 with the *TF*_1_-model, on the whole, *TF*_2_-model is the preferable model.

The above-mentioned information allows us to state that the proposed models correspond to the five generally mentioned recognized principles of constructing a QSPR/QSAR model. However, it seems appropriate to dwell on a number of features of the considered method.

A very useful feature of the approach under consideration is its significant heuristic potential due to the possibility of approximately formulating statistical hypotheses as follows:

- Whether (and if so, how much) the considered endpoint depends on the representation of molecules using SMILES;
- Whether (and if so, to what extent) the considered endpoint depends on the representation of molecules using graphs;
- Whether the representation of the molecular features extracted from SMILES and the graph provide a synergetic effect (i.e., improving the predictive potential of a model in the comparison of the separate cases considering the SMILES-based model and graph-based model);
- Whether *IIC* improves the predictive potential of models based on SMILES-based representation of molecules;
- Whether *IIC* improves the predictive potential of models based on a graph-based representation of molecules;
- Whether *CII* improves the predictive potential of models based on SMILES-based representation of molecules;
- Whether *CII* improves the predictive potential of models based on a graph-based representation of molecules;
- Whether the combined use of *IIC* and *CII* has a synergistic effect, that is, whether observed improvement of the predictive potential of models occurs if applying *IIC* and *CII* together compared to the cases of using *IIC* and *CII* separately.

In principle, the list of similar hypotheses that can be formulated and, accordingly, tested within the framework of the approach under consideration, can also be expanded. However, it seems more appropriate to consider the mentioned possibilities, providing them with brief explanations.

In fact, only a part of the hypotheses listed above is considered here. The results can be formulated as follows:

1. The combined use of correlation weighting of SMILES attributes and graph invariants improves the predictive potential of the hERG inhibition model expressed as *pIC*50;

2. For the considered compounds, the use of *CII* provides a better predictive potential than that of models built using *IIC*;

3. The observed statistical results for the three random splits of the available connections in the training and control sets are in good agreement with each other.

Are there valuable models? If there are “valuable” models, then there must be models that are not “valuable”. How to distinguish valuable models from not very valuable ones? It has been stated that “All models are wrong, but some are useful” [35]. Thus, how to distinguish useful models from a set of wrong ones? The reproducibility of results and their clarity (graphical representation [36]) are most likely the main features of the utility model. In this paper, for this purpose, attempts were made to build several models using different splits. The development of criteria for the predictive potential of models is also part of the research designed to identify useful models. In this paper, for this purpose, attempts were made to compare two new criteria for the predictive potential of the model, the *IIC* (*TF*_1_) and the *CII* (*TF*_2_).

One can extract two basic components in the total large variety of QSAR studies: (i) “applicative” studies and (ii) “theoretical” studies. “Applicative” studies aim to integrate the results of applying current approaches to solve practical tasks. “Theoretical” studies aim to attempt to develop new conceptions of the QSPR/QSAR analysis. This study contains both applicative and theoreatical parts. On the one hand, here, the Monte Carlo optimization technique described in the literature is aimed to build up (almost) standard models (applicative part). On the other hand, new criteria of the predictive potential are studied (theoretical part).

Thus, the epistemological aspect of the provided QSAR research, here, is presented in the form of confirmation of two statements. First, all QSAR models are random events if they are built using random distributions in training and validation sets. Second, the usefulness of random QSAR models can be stated if the variance in the values of statistical characteristics is acceptably small.

The Supplementary Materials section contains the technical details related to the described approach.

## 4. Methods

### 4.1. Data

The numerical data on 113 piperidine derivatives (pyridine-substituted piperidines, tertiary alcohol-bearing piperidines, spirocyclic piperidines, and isoxazole-containing piperidines) were taken from the literature [9]. The activity is expressed as −logIC50 or *pIC*50 [9]. The set of compounds is split into (i) active training (≈25%), (ii) passive training (≈25%), (iii) calibration (≈25%), and (iv) validation sets (≈25%). Each set has a defined task. The active training set is used to build the model; molecular features extracted from the simplified molecular-input line-entry system (SMILES—which represents the structure) [28,29,37], of the active training set are involved in the Monte Carlo optimization to provide correlation weights for the above features, which provide the largest target function value on the active training set. The passive training checks whether the model for the active training set is satisfactory for SMILES that were not involved in the active training set. The calibration set should detect when overtraining (overfitting) starts. The validation set provides the possibility to assess the predictive potential of a model since the data from the validation set is unknown while building up a model. Our experience with CORAL shows that equal distribution over the four sets mentioned is likely the most rational strategy.

At the beginning of the optimization, the correlation coefficients between the experimental values of the endpoint and the descriptor simultaneously increase for all sets, but the correlation coefficient for the calibration set reaches a maximum; this is the start of overtraining, and further optimization leads to a decrease of the correlation coefficient for the calibration set. Optimization should be stopped when overtraining starts. After stopping the Monte Carlo optimization procedure, the validation set is needed to assess the model’s predictive potential.

### 4.2. Optimal Descriptor 

The optimal descriptor, calculated with the representation of the molecular structure using the SMILES, 37serves as the basis of a model for cardiotoxicity. The optimal descriptor for the predictive model of the endpoint is calculated with Equation (7):

(7) $$pIC_{50}=C_{0}+C_{1}\times DCW\left(T,N\right)$$

(8) $$DCW\left(T,N\right)=\overset{NA}{\underset{k=1}{\sum }}CW(S_{k})+\overset{NA-1}{\underset{k=1}{\sum }}CW(SS_{k})\;$$

where *T* is an integer that separates molecular features extracted from SMILES into rare and non-rare ones. The non-rare features serve to build up the model. The rare features are not used to build up the model. *N* is the number of epochs in the optimization of the correlation weights. *Sk* is a SMILES atom, i.e., one SMILES line symbol (e.g., ‘=’, ‘O’) or a group of symbols that cannot be examined separately (e.g., ‘Cu’, ‘%11’). *SSk* is a couple of SMILES atoms. *CW*(*S_k_*) and *CW*(*SS_k_*) are the correlation weights of the SMILES attributes (*SAk*). *NA* is the number of non-rare SMILES attributes. 

The Supplementary Materials (Table S1) contains an example of the DCW(1, 15) calculation.

### 4.3. Monte Carlo Optimization

Equation (2) needs the numerical data on the above correlation weights. Monte Carlo optimization is employed to calculate the correlation weights. Here, two target functions for the Monte Carlo optimization are examined:

(9) $$TF_{0}=r_{AT}+r_{PT}-\left|r_{AT}-r_{PT}\right|\times 0.1$$

(10) $$TF_{1}=TF_{0}+IIC_{C}\times 0.5$$

(11) $$TF_{2}=TF_{1}+CII_{C}\times 0.5$$

Equation (3) is defined empirically during the development of many different models. Variables $r_{AT}$ and $r_{PT}$ are correlation coefficients between the observed and predicted values of the endpoint for the active training set and passive training set, respectively. *IIC_C_* is the index of ideality of correlation [28]. *IIC_C_* is calculated with data on the calibration set as follows:

(12) IICC=rCmin(MAEC−,MAEC+) max(MAEC−,MAEC+) 

(13) $$\min\left(x,y\right)=\left\{\begin{array}{c} x,\;if\;x<y\\ y,otherwise \end{array}\right.$$

(14) $$\max\left(x,y\right)=\left\{\begin{array}{c} x,\;if\;x>y\\ y,otherwise \end{array}\right.$$

(15) MAEC−=1N−∑Δk, N− is the number of Δk<0

(16) MAEC+=1N+∑Δk, N+ is the number of Δk≥0

(17) $$\Delta_{k}=observed_{k}-calculated_{k}$$

The corresponding values of the endpoint are observed and calculated. 

The correlation intensity index (*CII*), similar to the *IIC*, was developed to improve the quality of the Monte Carlo optimization used to build up QSPR/QSAR models. *CII* is calculated as follows:

(18) $$CII_{C}=1-\sum Protest_{k}$$

(19) $$Protest_{k}=\left\{\begin{array}{ll} R_{k}^{2}-R^{2}, & if\;R_{k}^{2}-R^{2}>0\\ \;0, & otherwise \end{array}\right.\;$$

where *R*^2^ is the correlation coefficient for a set that contains n substances. *R*^2^*_k_* is the correlation coefficient for *n* − 1 substances of a set after removing the *k*-th substance. If (*R*^2^*_k_* − *R*^2^) is larger than zero, the *k*-th substance is an “opponent” for the correlation between the experimental and predicted values of the set. A small sum of “protests” means a more “intense” correlation.

### 4.4. Applicability Domain

The described models’ applicability domain defines the “statistical defects” of molecular features extracted from SMILES or HSG. These are calculated as follows:

(20) $$d_{k}=\frac{\left|P(A_{k})-P\prime (A_{k})\right|}{N\left(A_{k}\right)+N\prime \left(A_{k}\right)}+\frac{\left|P(A_{k})-P\prime \prime (A_{k})\right|}{N\left(A_{k}\right)+N\prime \prime \left(A_{k}\right)}+\frac{\left|P\prime (A_{k})-P\prime \prime (A_{k})\right|}{N\prime \left(A_{k}\right)+N\prime \prime \left(A_{k}\right)}$$

where *P*(*A_k_*), *P*′(*A_k_*), and *P*″(*A_k_*) are the probability of *A_k_* in the active training, passive training, and calibration sets, respectively; *N*(*A_k_*), *N*′(*A_k_*), and *N*″(*A_k_*) are frequencies of *A_k_* in the active training, passive training, and calibration sets, respectively. The statistical SMILES defects (*D_j_*) are calculated as follows:

(21) $$D_{j}=\sum_{k=1}^{NA}d_{k}$$

where *NA* is the number of non-blocked SMILES attributes in the SMILES.

A SMILES falls in the applicability domain if

(22) $$Dj<2\bar{D}$$

where $\bar{D}$ is the average statistical defect on all compounds. 

## 5. Conclusions 

The suggested approach provides reliable cardiotoxicity models since their predictive potential is confirmed for three random splits into training and validation sets. The Monte Carlo optimization with the target function *TF*_2_ calculated with the correlation intensity index (Equation (11)) is more accurate and more reliable than optimization with the index of ideality of correlation, i.e., the target function *TF*_1_ (Equation (10)). Figure 4 illustrates the simplicity of applying the model for comparison of the potential biological activity of different molecules. In fact, such analysis can be a tool for the preliminary assessment of biological activity only on the basis of a set of Monte Carlo computation experiments with different distributions of available data in the training and validation (test) sets.

## Figures and Tables

**Table 1:** The statistical quality of the QSAR model for cardiotoxicity was obtained with Monte Carlo optimization with target function *TF*_1_.

	n	R^2^	CCC	IIC	CII	Q^2^	Q^2^_F1_	Q^2^_F2_	Q^2^_F3_	RMSE	MAE	F
A *	28	0.5552	0.7140	0.5588	0.7844	0.4940				0.424	0.371	32
P	28	0.6911	0.7400	0.6525	0.8394	0.6454				0.438	0.373	58
C	29	0.9101	0.9524	0.9536	0.9436	0.8955	0.9083	0.8998	0.9424	0.160	0.129	273
V	28	0.9146	-	-	-	-	-	-	-	-	0.20	0.15
A	29	0.8548	0.9217	0.8629	0.9300	0.8306				0.241	0.201	159
P	28	0.8893	0.8958	0.3877	0.9303	0.8740				0.284	0.222	209
C	28	0.8680	0.9261	0.8836	0.9273	0.8451	0.8346	0.8340	0.8658	0.242	0.194	171
V	28	0.8959	-	-	-	-	-	-	-	-	0.27	0.24
A	28	0.5347	0.6968	0.7312	0.7836	0.4239				0.436	0.379	30
P	28	0.5607	0.7087	0.4930	0.7714	0.4976				0.451	0.387	33
C	28	0.8374	0.9124	0.9150	0.8940	0.8173	0.8201	0.8192	0.8808	0.228	0.179	134
V	29	0.9181	-	-	-	-	-	-	-	-	0.21	0.16

* A = active training set; P = passive training set; C = calibration set; V = validation set.

**Table 2:** The statistical quality of the QSAR model for cardiotoxicity was obtained with Monte Carlo optimization with target function *TF*_2_.

	n	R^2^	CCC	IIC	CII	Q^2^	Q^2^_F1_	Q^2^_F2_	Q^2^_F3_	RMSE	MAE	F
A	28	0.5067	0.6726	0.7118	0.7752	0.4439				0.446	0.386	27
P	28	0.6341	0.6605	0.6043	0.8438	0.5801				0.481	0.436	45
C	29	0.9143	0.9556	0.9562	0.9494	0.9011	0.9206	0.9132	0.9501	0.149	0.123	288
V	28	0.9347	-	-	-	-	-	-	-	-	0.19	0.15
A	29	0.5253	0.6887	0.6764	0.7989	0.4496				0.436	0.387	30
P	28	0.6806	0.7591	0.8009	0.8318	0.6362				0.395	0.354	55
C	28	0.9494	0.9717	0.9741	0.9742	0.9396	0.9475	0.9473	0.9574	0.136	0.105	488
V	28	0.9292	-	-	-	-	-	-	-	-	0.19	0.16
A	28	0.5870	0.7398	0.7662	0.7886	0.5004				0.410	0.361	37
P	28	0.6347	0.7708	0.5116	0.7931	0.5878				0.412	0.341	45
C	28	0.8773	0.9322	0.9366	0.9231	0.8587	0.8548	0.8540	0.9038	0.205	0.174	186
V	29	0.9255	-	-	-	-	-	-	-	-	0.22	0.18

**Table 3:** Promoters of an increase or decrease of cardiotoxicity (*pIC*50) that were observed for computational experiments with split-1.

ID	SAk	CWs Run1	CWs Run2	CWs Run3	NA *	NP	NC	dk
	Promoters of increase							
1	(...........	0.1947	0.7446	0.5440	28	28	29	0.0000
2	1...........	0.7284	0.4211	0.3577	28	28	29	0.0000
3	O...(.......	0.3798	0.9650	0.5790	28	28	29	0.0000
4	c...........	0.1359	0.7877	0.3057	28	28	29	0.0000
5	c...1.......	0.6071	0.0798	0.1767	28	27	28	0.0009
6	c...c.......	0.6871	0.6369	0.6244	28	28	29	0.0000
7	C...1.......	0.2662	0.3189	0.4394	25	23	28	0.0038
8	1...(.......	1.5716	0.9427	0.9525	24	19	26	0.0063
9	N...(.......	0.5049	0.6520	0.3610	24	20	23	0.0043
10	2...........	0.3701	0.3364	0.3724	20	20	16	0.0058
11	F...(.......	0.4099	0.3220	0.1576	17	24	18	0.0085
12	c...F.......	0.2340	0.7688	0.0176	13	14	9	0.0105
13	n...(.......	0.4261	0.7984	0.6231	12	10	12	0.0042
14	C...=.......	1.2645	0.4920	0.8643	11	5	6	0.0195
15	F...1.......	0.0991	0.3553	0.9094	11	11	9	0.0053
	Promoters of decrease							
1	C...........	−0.0788	−0.0409	−0.0820	28	28	29	0.0000
2	=...(.......	−0.6876	−0.8715	−0.2888	26	26	28	0.0009
3	O...=.......	−1.0322	−0.3332	−0.3444	26	26	28	0.0009
4	O...C.......	−0.4151	−0.2547	−0.0993	17	12	19	0.0094
5	c...C.......	−1.3073	−0.8511	−0.1159	17	16	15	0.0037

* *NA*, *NP*, and *NC* are the frequencies of a molecular feature in the active training, passive training, and calibration sets, respectively.

**Table 4:** The comparison of QSAR models for cardiotoxicity as suggested in the literature.

N Train	R^2^ Train	Q^2^ Train	RMSE Train	N Valid	R^2^ Valid	RMSE Valid	Reference
113	0.6576 0.6264 0.6872	0.6341 0.5801 0.6516	-	-	-	-	[9] MOE-models
113	0.6600 0.6896 0.7498	0.6272 0.6565 0.7118	-	-	-	-	[9] MACCS-models
623	0.29	-	0.630	345	0.41	0.550	[30]
309	0.911	-	0.264	112	0.860	0.301	[33]
4081	0.46	-	0.59	-	-	-	[34]
85 85 84	0.6774 0.8463 0.6169	0.6641 0.8379 0.5991	0.358 0.253 0.380	28 28 29	0.9146 0.8959 0.9181	0.203 0.275 0.210	Split-1 Split-2 Split-3 (In this work, *TF*_1_)
85 85 84	0.6348 0.7056 0.6746	0.6201 0.6931 0.6596	0.382 0.346 0.352	28 28 29	0.9347 0.9292 0.9255	0.189 0.186 0.221	Split-1 Split-2 Split-3 (In this work, *TF*_2_)

## Data Availability

Data available within the article or its Supplementary Materials.

## Supplementary Materials

The following supporting information can be downloaded at: https://www.mdpi.com/article/10.3390/molecules28186587/s1, Supplementary Materials section contains an example of the *DCW*(1, 15) calculation with Equation (1) for the *TF*_1_-model (split-1, Table S1) and details on the *TF*_2_-model calculated with Equation (4) (split-1, Table S2).
